# Comparison of the Effects of 5-Hydroxymethylfurfural in Milk Powder Matrix and Standard Water on Oxidative Stress System of Zebrafish

**DOI:** 10.3390/foods11121814

**Published:** 2022-06-20

**Authors:** Yingyu Hou, Xinyue Zhang, Xixia Liu, Qin Wu, Jianjun Hou, Ping Su, Qian Guo

**Affiliations:** 1 Hubei Key Laboratory of Edible Wild Plants Conservation and Utilization, Hubei Normal University, Huangshi 435002, China; hyyfjm2022@163.com (Y.H.); zxyue2022@126.com (X.Z.); wuqin20200106@163.com (Q.W.); jjhou@mail.hzau.edu.cn (J.H.); suping1202@126.com (P.S.); gq977643869@163.com (Q.G.); 2 Hubei Engineering Research Center of Special Wild Vegetables Breeding and Comprehensive Utilization Technology, Hubei Normal University, Huangshi 435002, China

**Keywords:** furfural compounds, dairy products, zebrafish embryo, toxicity, antioxidant enzyme

## Abstract

5-Hydroxymethylfurfural (5-HMF) and furfural (FF) are products of the maillard reaction (MR) in milk powder and their safety is controversial. The concentration changes of 5-HMF and FF after a period of cold storage were determined by high-performance liquid chromatography (HPLC). Then, we compared the toxicity effects of 5-HMF (2, 20, or 200 μM) in milk powder matrix and standard water on the oxidative stress system of zebrafish embryos. The results showed that the concentration of 5-HMF was stable, and the concentration of FF degraded over time. 5-HMF-exposed zebrafish embryos had a LC_50_ value of 961 μM for 120 h. High-concentration of 5-HMF exposure resulted in developmental toxicity and induced oxidative stress. 5-HMF exposure resulted in low expression of *gstr* gene at 200 μM in both matrices. Moreover, *sod*, *cat*, *gstr*, and *gpxla* genes were differentially highly expressed in other groups or showed no significant difference. Residual levels in all groups were well below the exposed dose, with a maximum value of only 0.4‰. These results provided a theoretical basis for understanding the effects of 5-HMF exposure in milk powder matrix on the oxidative stress system and suggested that the presence of 5-HMF in our daily consumption of milk powder does not produce significant toxic effects and need not be overstressed.

## 1. Introduction

5-hydroxymethylfurfural (5-HMF) and furfural (FF) are small molecules produced by maillard reaction (MR) and the caramelization of many sugary foods during heat treatment [1]. Many factors in the food processing environment, such as pH value, pressure, temperature, and food composition, affect the production and reaction process of 5-HMF and FF [2]. Milk powder is made from fresh milk by evaporation and spray drying. The preparation temperature can be as high as 137 °C [3]. The reducing sugars, amino acids, and proteins contained in it are easily produce a certain amount of MR products including 5-HMF and FF.

The European Food Safety Authority (EFSA) has established an acceptable daily intake value of 0.5 mg/kg body weight for FF and considers the maximum use limit of 5-HMF in dairy products as 15 mg/kg [4]. The benefit or harm of 5-HMF to human health is still controversial. Studies have shown that 5-HMF has pharmacological activities, such as antioxidation [5], anti-inflammatory [6], and anti-tumor [7]. Moreover, it also produces nephrotoxicity [8], potential genotoxicity [9,10] and so on. According to these reports, the toxicity of 5-HMF was studied with a high dose in buffer. It is unknown whether the 5-HMF is beneficial or harmful to human health in a food matrix with a dietary exposure dose.

Researchers have established a variety of methods to detect the concentration of FF and 5-HMF in foods [11,12,13]. Cui et al. [14] detected 55 dairy product samples, and the concentration of 5-HMF in most milk powder samples was about 10 times higher than FF. The maximal concentration of 5-HMF was 0.624 mg/kg. Although the residues of 5-HMF in milk samples are lower than the residue limits in state criteria for food safety, it is worth studying the toxicity differences with the dietary exposure dose in milk matrix and standard water. Oxidative stress is a phenomenon caused by the imbalance between the production and accumulation of reactive oxygen species (ROS) in cells, tissues and the ability of biological systems to detoxify reaction products. Excessive ROS from endogenous or exogenous sources plays a role in many diseases [15]. Antioxidants prevent ROS-induced damage by preventing the formation of ROS, removing them, or promoting their decomposition [16]. Therefore, it is very important to understand the role of small molecular compounds in food on the human oxidative stress system. From the previous two reports, we know that 5-HMF has antioxidant activity [5] and induces ROS production [17], but has an inconsistent conclusion. Therefore, the potential effects of daily intake of 5-HMF from milk matrix and standard water on the oxidative stress system is worth clarifying.

The zebrafish is an emerging model creature that is gradually widely used in the field of toxicology. It has been sequenced and annotated to reveal a large degree of evolutionary conservatism [18], and its anatomical and physiological characteristics are highly homologous to human [19]. Compared with rodent model animals, zebrafish have a shorter life cycle and stronger reproductive capacity. Zebrafish embryos can be used as biosensors to quickly detect the biological activity and toxicity of a large number of chemicals [20]. Jiang et al. [17] using zebrafish embryo-related experiments found that a high concentration of 5-HMF induced the production of ROS, resulting in developmental toxicity. However, the research was conducted with a high concentration of 5-HMF, which did not reflect the impact of daily intake on the human health.

In this study, the degradation characteristics of 5-HMF and FF over time were identified using high-performance liquid chromatography (HPLC). The oxidative stress toxicity of different concentrations of 5-HMF in milk powder matrix and standard water were carefully studied using zebrafish embryos as a model animal, and then the residue of 5-HMF in zebrafish larvae was detected to explore the effect of milk powder matrix and standard water on its accumulation in vivo. The aim of this study is to provide a reference for studying the effects of actual dietary intake dose of 5-HMF on human health and provide data support for further defining the residue limit and toxic concentration of 5-HMF.

## 2. Materials and Methods

### 2.1. Materials

Milk powder was purchased from Meiyijia supermarket; 5-HMF and FF standard were purchased from TMRM (Beijing, China). RNAiso Plus reverse transcriptase kit and fluorescence quantitative kit were purchased from Takara (Beijing, China); Primers were synthesized by Sangon Biotech (Shanghai, China). Superoxide dismutase (SOD), glutathione (GSH), and malondialdehyde (MDA) detection kit were purchased from COMIN (Suzhou, China). ROS detection kit was purchased from Nanjing Jiancheng Bioengineering Institute (Nanjing, China). Other chemicals were purchased from Sinopharm Chemical Reagent Co., Ltd. (Shanghai, China).

### 2.2. Determination of the Degradation Characteristics of 5-HMF and FF over Time by HPLC

The separation column was a Diamonsil Plus 5 μm C18 150 × 4.6 mm in isocratic mode with ultraviolet detection under 284 nm by HPLC (Agilent 1260 Infinity, Santa Clara, CA, USA). The mobile phase was acetonitrile–water (volume ratio was 15:85); the column temperature was 25 °C; the flow rate was 1.0 mL/min; the single injection volume was 5 μL. The 5-HMF and FF standards with a concentration of 1 M were prepared as mother solution in phosphate buffered saline (PBS). Then, 5-HMF and FF with equal volumes were mixed to prepare series of 5-HMF and FF standards (5, 10, 20, 40, 80, 160, 320, or 500 μM). The samples with 5-HMF and FF concentrations of 80, 160, or 320 μM in buffer were stored at 4 °C, and the final concentrations of 5-HMF and FF were detected at intervals of 1, 2, 4, 7, 12, and 21 days. After analyzing the relationship between 5-HMF, FF, and peak area, we identified the degradation characteristics of 5-HMF and FF over time.

### 2.3. 5-HMF Exposure and Developmental Observations

Adult AB-strain zebrafish were maintained in a flow-through system with a 14:10 light/dark period and fed with freshly hatched brine shrimp three times daily. Adult male and female zebrafish (male/female ratio of 1/2) were paired in spawning containers overnight, and spawning was initiated with the light cycle. The embryos were placed in standard water (per 100 mL containing 6.48 mg NaHCO_3_, 0.56 mg KCl, 22.20 mg CaCl_2_, and 12.32 mg MgSO_4_·7H_2_O) and kept in a 28 °C light-controlled incubator.

Healthy zebrafish embryos at 6 h post fertilization (hpf) were randomly dispensed in 6-well plates at 28 embryos/well with 3 mL standard water and then exposed to series concentrations of 5-HMF (0, 6.25, 100, 400, 1600, or 3200 μM). All of experimental embryos were cultured in an incubator at 28 °C; 2 mL of solution was replaced daily in every well. The body length, mortality, and deformity rate were recorded at 120 h, and the deformities were observed under a stereoscopic microscope. The Probit model of IBM SPSS Statistics 26 (Armonk, NY, USA) software was used to calculate the LC_50_ value of 120 h.

The different concentrations of 3 mL milk powder matrix (blank control, 0.01%, 0.1%, 0.1%, 0.5%, and 1%) were exposed to zebrafish embryos, and the solution of milk powder matrix was changed every 24 h. The survival rate, hatching rate, and body length were recorded at 120 h, and the deformities were observed under stereoscopic microscope. According to the LC_50_ value and the residue limit of 5-HMF, 8 groups were designed with different concentrations (blank control, 2, 20, and 200 μM) in standard water and milk powder substrate 0.01%), respectively. There were 6 holes in each group and 30 embryos in each hole, and all exposure solutions are replaced every 24 h to maintain the original concentration. Exposure at 120 h was used for follow-up detection.

### 2.4. Determination of Oxidation Parameters

After 120 h of exposure, 60 zebrafish larvae were randomly selected from each group and divided into three parts on average. The zebrafish larvae were washed twice with PBS and dried. According to the operation instructions of the protein concentration, SOD, GSH, and MDA kit, the corresponding homogenate medium was added and homogenized in ice bath. Then, the collected supernatant samples were used to determine the data of SOD, GSH, MDA, and protein concentrations.

### 2.5. ROS Assay

After 144 h of exposure, 20 zebrafish larvae in each group were washed with PBS and dried. Ten μM fluorescent probes were added and incubated for 30 min according to the instructions of ROS determination kit. The zebrafish larvae were washed as cleanly as possible with PBS, and the fluorescence intensity was observed under fluorescence microscope. Meanwhile, 100 μL PBS was added to the rest of zebrafish larvae, then homogenized in ice bath, and centrifuged (4 °C 15,984× *g* for 10 min) to get the supernatant. After diluting 60 times, the fluorescence intensity of each group was detected in an i3X plate reader instrument.

### 2.6. Gene Expression Analysis

In each group, 20 zebrafish larvae were randomly collected after 120 h exposure and frozen at −80 °C for gene expression analysis. Total RNA was extracted from each group using RNAiso Plus. The cDNA was synthesized via reverse transcription according to the manufacturer’s protocols. The primers used in this study are shown in Table 1, and the real-time quantitative PCR procedure was set at 95 °C 30 s, 95 °C for 5 s, and 60 °C for 30 s, for 40 cycles. The expression level of the target gene was calculated by the 2^−ΔΔCt^ method.

### 2.7. Determination of 5-HMF Residues in Zebrafish Larvae

The 5-HMF residues in zebrafish larvae were detected using HPLC. Twenty zebrafish larvae in each group were washed with ultra-pure water 3 times. Then, 400 μL of 5-HMF (50 μM) was added to each tube and homogenized in ice bath. The sample was centrifuged for 5 min at 4 °C 15,984× *g*. The supernatant was filtered with a 0.22-μm filter membrane and detected by HPLC. The control group samples cultured in standard water were taken as the standard curve. The amount injected was 1, 2, 4, 8, 16, and 32 μL; the corresponding content was 50, 100, 200, 400, 800, 1600 pmol, respectively. The injection volume of the other samples was 16 μL, and the 5-HMF residual amount of each group of samples was calculated by subtracting the background concentration.

### 2.8. Statistical Analysis

All data were represented by a mean ± standard error. Statistical analysis was adopted IBM SPSS Statistics 26. The statistical difference between the exposure group and the control group was determined using one-way analysis of variance (ANOVA) followed by Dunnett’s post-test. The probability levels determined for all analyses were considered statistically significant at *p* < 0.05.

## 3. Results

### 3.1. Determination of the Degradation Characteristics of 5-HMF and FF over Time by HPLC

**Figure 1:**
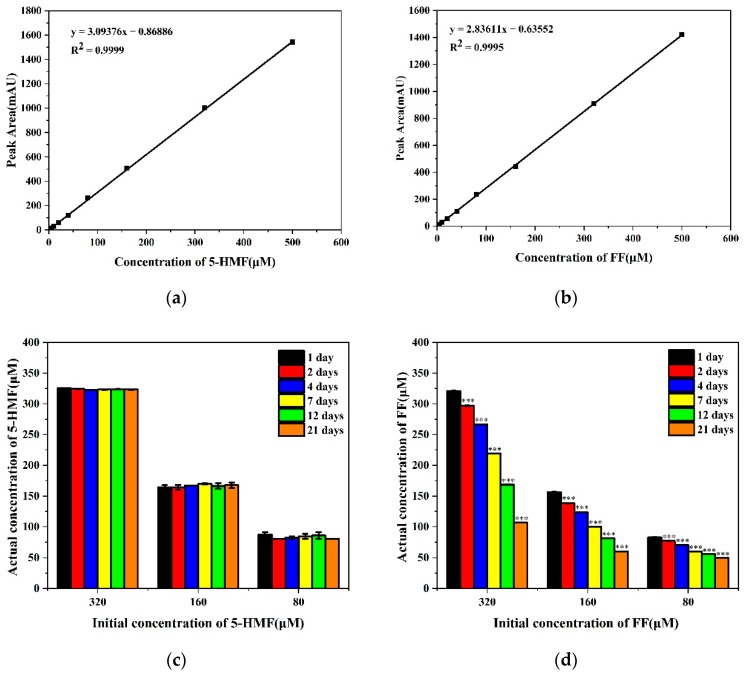
Concentration changes in 5-HMF and FF detected by HPLC. (**a**) Standard curve of 5-HMF; (**b**) Standard curve of FF; (**c**) Variation of 5-HMF at different concentrations with time; (**d**) Variation of FF at different concentrations with time. *** *p* < 0.001 vs. 1 day.

Linear regression was performed between concentration of 5-HMF, FF, and the peak area, respectively. The obtained regression equation: (1) y = 3.09376x − 0.86886, R^2^ = 0.9999 (5-HMF); (2) y = 2.83611x − 0.63552, R^2^ = 0.9995 (FF). The standard curve indicates that there was a good linear relationship between the concentrations and the peak area (Figure 1a,b). The concentration of 5-HMF is not obviously changed after 21 days, but the concentration of FF gradually decreased with the extension of the storage time (Figure 1c,d). Therefore, our subsequent experiments focused on 5-HMF.

### 3.2. The Determination of LD_50_ and Developmental Observations

**Figure 2:**
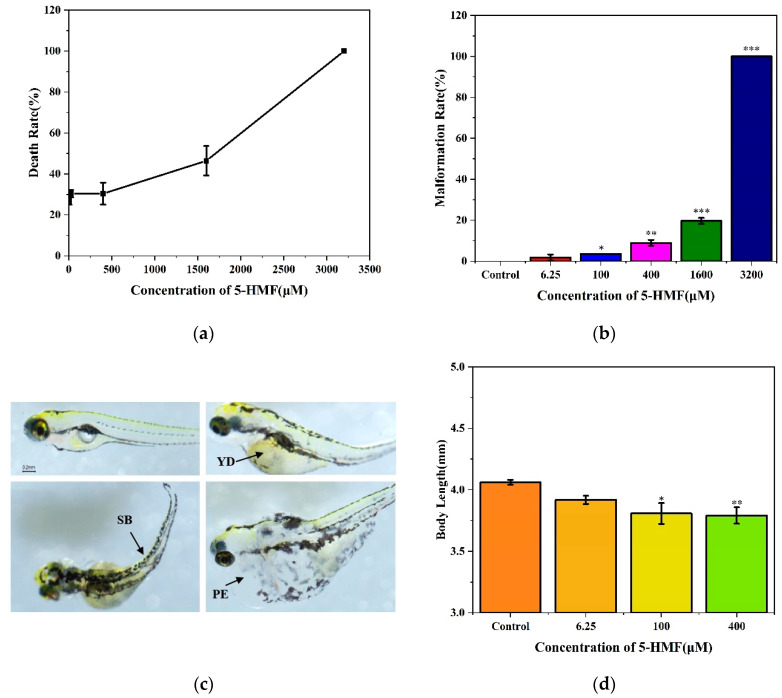
Developmental toxicity and teratogenicity of zebrafish larvae exposed to different concentrations of 5-HMF for 120 h. (**a**) Death rate of zebrafish embryos treated with different concentrations of 5-HMF; (**b**) Malformation rate of zebrafish embryos treated with different concentrations of 5-HMF; (**c**) Malformation of zebrafish larvae treated with high concentrations of 5-HMF, abbreviations: PE, pericardial edema; SB, spinal bending; YD, delayed absorption of yolk sac; (**d**) Body length of zebrafish larvae treated with different concentrations of 5-HMF. * *p* < 0.05, ** *p* < 0.01 and *** *p* < 0.001 vs. control.

We detected the development of zebrafish embryos exposed to different concentrations of 5-HMF for 120 h. At the concentration of 3200 μM, all the zebrafish larvae died and deformed. At the same time, the mortality and deformity rate of zebrafish embryos increased with the increase in exposure concentration in a concentration-dependent manner (Figure 2a,b). The LC_50_ value of zebrafish embryos exposed to 5-HMF was 961 μM for 120 h, and the 95% confidence interval was [656,1365]. High concentrations of 5-HMF exposure can cause developmental toxicity to zebrafish embryos, resulting in spinal bending, pericardial edema, and delayed absorption of yolk sac (Figure 2c). In addition, the body length growth of zebrafish larvae was inhibited in the 100 and 400 μM groups, and they had relatively few deformities (Figure 2d).

### 3.3. Suitable Exposure Concentration of Milk Powder Matrix

**Figure 3:**
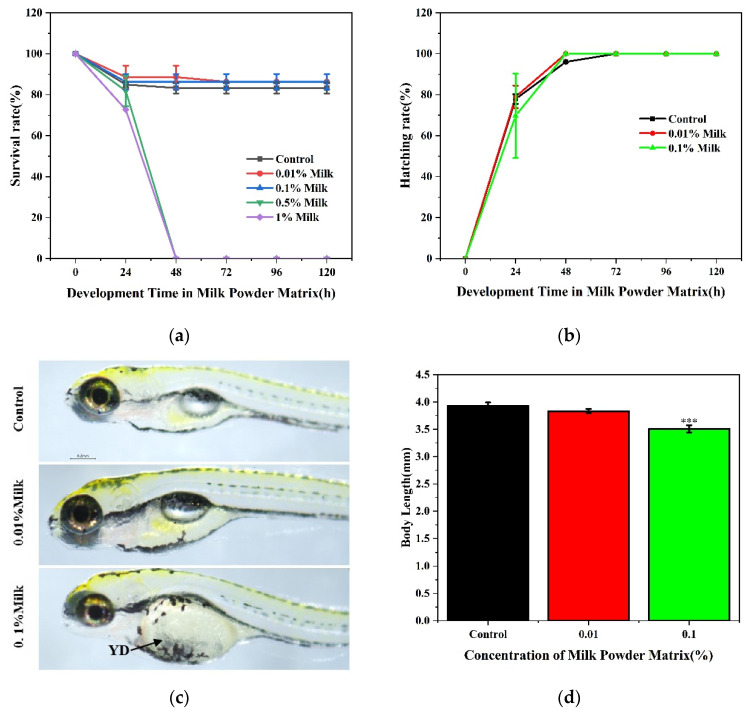
Effect of different concentrations of milk powder matrix incubation for 120 h on zebrafish larvae. (**a**) Survival rate of zebrafish embryos cultured with different concentrations of milk powder matrix; (**b**) Hatching rate of zebrafish embryos cultured with different concentrations of milk powder matrix; (**c**) Development of zebrafish larvae cultured with low concentration milk powder matrix; (**d**) Body length of zebrafish larvae cultured with low concentration of milk powder matrix. Abbreviations: Milk, milk powder matrix. *** *p* < 0.001 vs. control.

In order to explore the tolerance of zebrafish embryos to milk powder matrix, we set four concentrations of 0.01%, 0.1%, 0.5%, and 1%, and cultured 6 hpf zebrafish embryos for 120 h. At 48 h, zebrafish embryos could not survive normally in 0.5% and 1% milk matrix, while there was no significant difference in survival rate at other concentrations (Figure 3a). Zebrafish embryos could hatch faster in 0.01% and 0.1% milk matrix with a hatching rate of 100% in 48 h, while the control group could not hatch completely until 72 h (Figure 3b). Compared with the control group, the morphology and body length of zebrafish larvae in the 0.01% concentration group were not significantly different, while 0.1% showed delayed absorption of yolk sac and shorter body length (Figure 3c,d). In order to avoid milk powder matrix affecting the normal development of zebrafish embryos, we chose 0.01% milk powder matrix for follow-up experiments.

### 3.4. Determination of Oxidation Parameters

**Figure 4:**
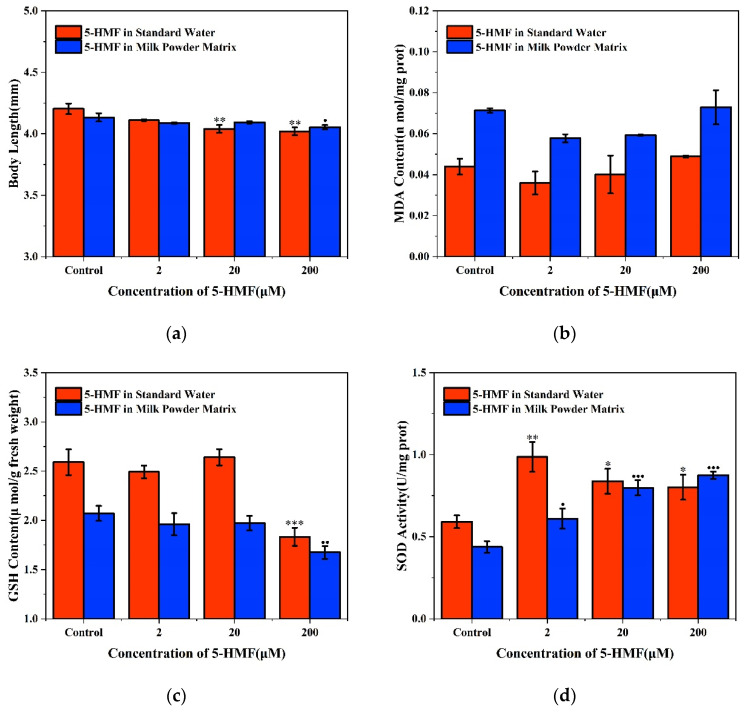
Effect of high, medium and low concentrations of 5-HMF on zebrafish embryos exposed to different matrices for 120 h. (**a**) Effect of 5-HMF in different matrices on the body length of zebrafish larvae; (**b**) Effect of different concentrations of 5-HMF in two matrices on the MDA content of zebrafish embryos; (**c**) Effect of different concentrations of 5-HMF on GSH content of zebrafish embryos in two matrices; (**d**) Effect of different concentrations of 5-HMF in two matrices on SOD activity of zebrafish embryos. * or ● *p* < 0.05, ** or ●● *p* < 0.01 and *** or ●●● *p* < 0.001 vs. control.

Zebrafish embryos were exposed to 5-HMF for 120 h. In standard water, the two high-concentration groups (20 μM and 200 μM) resulted in shortened body lengths of zebrafish larvae in a concentration-dependent manner. However, in the milk powder matrix, only one group with a concentration of 200 μM resulted in the shortest body lengths (Figure 4a). In the two matrices of standard water and milk powder, the concentration of MDA in zebrafish larvae was slightly lower than that of the control group when they were exposed to 2 μM and 20 μM 5-HMF, while it was higher than that of the control group with the concentration of 5-HMF at 200 μM. In general, the concentration of MDA in zebrafish larvae exposed to 5-HMF in milk powder matrix was higher than that in standard water (Figure 4b). The concentration of GSH was significantly lower in both matrices when concentration of 5-HMF was 200 μM, and the changes in GSH at 2 μM and 20 μM groups were not significantly different (Figure 4c). In standard water, SOD activity was significantly increased at 2 μM group, and it was no significant difference in the elevated 20 μM and 200 μM groups. In the milk powder matrix, SOD activity gradually enhanced with increasing concentrations, all of which were significantly different (Figure 4d).

### 3.5. ROS Assay

**Figure 5:**
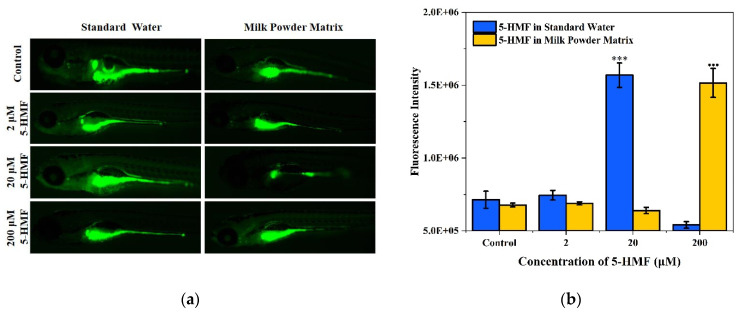
Induction of ROS production by medium and high concentrations of 5-HMF in different matrices. (**a**) Imaging of ROS staining in zebrafish larvae in different matrices; (**b**) Fluorescence intensity of zebrafish larvae ROS in different matrices. *** or ●●● *p* < 0.001 vs. control.

To compare the effects of high, medium, and low concentrations (200 μM, 20 μM, and 2 μM) of 5-HMF in two matrices on ROS production in zebrafish larvae. After 144 h of exposure, the results of ROS staining in zebrafish larvae (Figure 5) showed that 5-HMF (20 μM) induced a significant increase in ROS production in the standard water group. A 200 μM concentration of 5-HMF induced a significant increase in ROS production in the milk powder matrix. In addition, in the groups (control, 2 μM, and 20 μM), the ROS production was higher in standard water than that in milk powder matrix. Therefore, the low concentration of 5-HMF could protect the zebrafish larvae against oxidative damage in milk powder matrix.

### 3.6. Gene Expression Analysis

**Figure 6:**
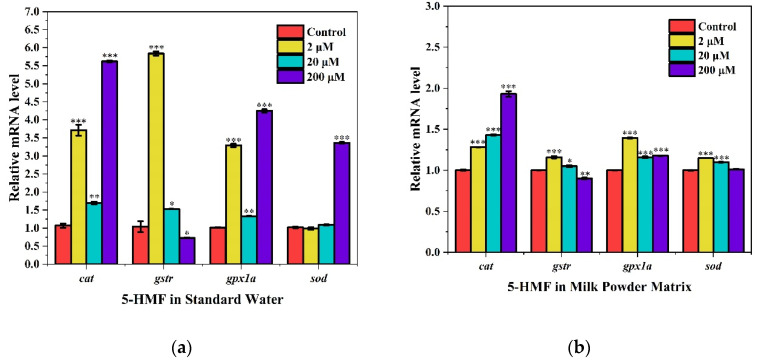
High, medium and low concentrations of 5-HMF in different matrices result in different degrees of altered gene expression of antioxidant systems. (**a**) Gene expression changes caused by 5-HMF in standard water; (**b**) Gene expression changes caused by 5-HMF in milk powder matrix. * *p* < 0.05, ** *p* < 0.01 and *** *p* < 0.001 vs. control.

The RT-qPCR analysis of antioxidant enzyme system-related genes is shown in Figure 6. In standard water, *cat* and *gpxla* genes were highly expressed in all three concentration groups compared with the control group, and the 200 μM group showed the highest upregulation level, while the 20 μM group showed the least change in upregulation level compared with the other two concentrations. The *gstr* gene was highly significantly upregulated in the 2 μM group, significantly highly expressed in the 20 μM group, and lowly expressed in the 200 μM group. The *sod* gene was upregulated only at 200 μM, and the other groups were not significantly different from the control group. In the milk powder matrix, *cat* gene expression increased with increasing 5-HMF concentration. The expression of *gstr*, *gpxla*, and *sod* were all most significantly increased in the 2 μM concentration group. They were also expressed upregulated in the 20 μM concentration group. The *gstr* expression was downregulated and *gpxla* expression was upregulated in the concentration of 200 μM group; *sod* expression was not significantly different from the control group.

### 3.7. Determination of 5-HMF Residues in Zebrafish Larvae

**Figure 7:**
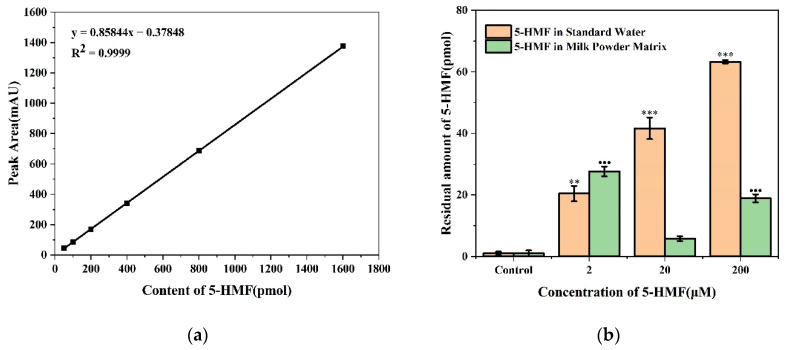
HPLC detection of 5-HMF residues in each group of zebrafish larvae after 120 h exposure. (**a**) The standard curve of 5-HMF after the addition of standard samples; (**b**) The residues of 5-HMF in different matrices for each concentration group. ** *p* < 0.01, *** or ●●● *p* < 0.001 vs. control.

The content of 5-HMF was used as the abscissa and the peak area as the ordinate. The regression equation was y = 0.85844x − 0.37848 with R^2^ = 0.9999, which shows a good linear relationship (Figure 7a). As can be seen from Figure 7b, the 5-HMF residue in zebrafish larvae increased in a dose-dependent manner in standard water. But in the milk powder matrix, the performance was different. The residue of 5-HMF was the highest in the 2 μM group, followed by the 200 μM group. The residue level in the 200 μM group was about 1/3 of that in the standard water. The residue levels in the 20 μM group were not significantly different from those in the control group, and the residue levels were much lower in all groups compared with the exposure dose, with a maximum of only 0.4‰.

## 4. Discussion

The MR products FF and 5-HMF are widely present in foods. In particular, 5-HMF has both pharmacological activity and toxic effects, which are related to its concentration, reaction matrix, and metabolites [2,21], and its safety remains controversial. Milk powder and other dairy products are commonly in demand in our daily life, especially by infants and adolescents during their growth, and have high quality requirements. FF and 5-HMF have been detected to varying degrees in various milk powders, and the levels of 5-HMF are high [14]. Therefore, it is important to investigate whether daily exposure concentrations of 5-HMF in milk powder matrix have potential toxic effects on humans. In this study, the effects of daily exposure concentrations and higher concentrations of 5-HMF in both standard water and milk powder matrix on the oxidative stress system were compared in zebrafish embryos. The results showed that concentrations of 5-HMF did not change over time, but concentrations of FF decreased by 50% after 21 days of storage at 4 °C. Exposure to high concentrations of 5-HMF resulted in developmental toxicity and induced oxidative stress. Low concentrations of 5-HMF in milk powder matrix had no significant effect on oxidative stress in zebrafish embryos. Moreover, the milk powder matrix had some mitigating protection against high concentrations of 5-HMF toxicity, such as reduction in body length, production of ROS, and alteration in gene expression.

During heat treatment, 5-HMF is formed mainly by MR [22] or lactose isomerization and subsequent degradation reactions [23], which can be used as a potential indicator to evaluate the severity of heat treatment during food processing [24]. Furfural is also formed by MR, direct cleavage of pentose, and indirect conversion from pentosan [25]. In this study, we found that the concentration of FF gradually decreased over time at 4 °C, but the concentration of 5-HMF did not change. Shen et al. [26] analyzed 36 samples covering the entire infant formula production chain and found the largest increase of 5-HMF in the spray drying (6 to 11 times) and homogenization stages (12 to 33 times) with a high hazard quotient value (3.11). In the study by Cui et al. [14], 5-HMF was detected in most milk powder samples at higher levels than FF. Therefore, we speculated that FF may degrade on its own or be transformed into other products, which are not discussed much in this paper. The 5-HMF is the potentially hazardous substance in dairy products that people are exposed to on daily intake. Thus, it is of real significance to clarify the toxicity of 5-HMF according to our daily intake dose.

High concentrations of 5-HMF cause developmental toxicity and induce oxidative stress, while low concentrations of 5-HMF promote SOD activity. The toxic effects of 5-HMF are somewhat dose-dependent. Oxidative stress occurs when the production of ROS is increased, or the antioxidant defense of the body is reduced. The concentration of MDA indicates the level of lipid peroxidation, and GSH and SOD are antioxidants used to mitigate ROS damage [27]. In this study, zebrafish embryos showed increasing mortality and malformation rate with increasing concentration of 5-HMF. Especially, the high concentration of 5-HMF reduced the body length of zebrafish larvae and induced spinal curvature, pericardial edema, and delayed yolk sac uptake. At concentrations of 20 or 200 μM in different matrices, 5-HMF also induced a significant increase in ROS. These were consistent with the high concentration of 5-HMF induced developmental toxicity and increased ROS levels in zebrafish larvae as reported by Jiang et al. [17]. Wang et al. [28] found that 100 μM of 5-HMF treatments of PC12 and HT22 cells significantly increased ROS levels and induced anxiety and depression-like behavior in adolescent mice at gavage doses of 0.15 and 1.5 mg/kg in vivo. We also found that 200 μM of 5-HMF induced an increase in MDA content and a decrease in GSH content in either substrate, possibly inducing oxidative stress. To combine with the daily intake, we further reduced the experimental concentration according to the residue limit to simulate the 5-HMF dose in conventional milk powder intake. We found that general intake of 2 μM concentration did not cause significant changes in MDA, GSH, and ROS contents and improved SOD activity. Wang et al. [29] found that 5-HMF at 0.79 μM was protective against H_2_O_2_-induced oxidative damage in hepatocytes, maintained cell morphology, and inhibited caspase-9 and caspase-3 expression. *cat*, *gstr*, *gpxla* and, *sod* are antioxidant enzyme lineage related genes in zebrafish [30,31]. In this study, *cat*, *gstr*, and *gpxla* were significantly upregulated at 2 μM groups, indicating a possible stronger promotion of antioxidant activity. The level of gene upregulation at 20 μM groups was mostly inferior to that at the 2 μM groups, indicating a reduced promotion effect. The change of *sod* expression in standard water at 20 μM group was no significance, which might be the reason why the increase in ROS induced by 5-HMF. At 200 μM groups, the downregulation of *gstr* may have led to a weakening of the antioxidant effect of the organism, while the expression of *sod* in the milk powder matrix was not significantly from that of the control group. These may be related to the disorder of the antioxidant system due to the surge of ROS. The above results suggest that high concentrations of 5-HMF may induce oxidative stress and low concentrations would promote antioxidant activity. However, further experiments are needed to clarify the concentration range and explore the mechanism.

After exploring the adaptation concentration of milk powder matrix, we found that 0.01% milk powder matrix could allow complete hatching of zebrafish embryos earlier and not affect their growth and development. It has a mitigating protective effect against the harm caused by high concentrations of 5-HMF. Casein is the main protein in milk and has the ability to scavenge free radicals [32,33]. Zunquin et al. [34] found that casein significantly reduced iron-induced lipid peroxidation and prevented the deleterious effects of iron overload on tissues. Our results suggested that the milk powder matrix had a mitigating effect on the damage caused by 5-HMF and maintained organismal stability.

The residue concentration in zebrafish larvae was too small to detect 5-HMF directly by HPLC. Thus, we tried to detect its concentration using a spiking method. Our results showed that the residue dose was much lower in all groups compared to the exposure dose, indicating the 5-HMF did not accumulate in zebrafish larvae. This result implied that the 5-HMF may be a low accumulative small molecular substance.

## 5. Conclusions

Our finding suggested that 5-HMF is more stable than FF in the production and storage of milk powder, and its generation needs to be strictly controlled. High concentrations of 5-HMF exposure resulted in developmental toxicity and induced oxidative stress. The milk powder matrix promoted the hatching of zebrafish embryos and provided some mitigating protection against the changes in body length, ROS, and gene expression caused by high concentrations of 5-HMF. These results provided a theoretical basis for understanding the effects of 5-HMF exposure in milk powder matrix on oxidative stress systems. This study demonstrates that 5-HMF causes significant damage only at high concentrations. In addition, the 5-HMF content detected in milk powder for daily consumption does not induce significant oxidative stress or developmental toxicity, and consumers need not be overstressed in this regard.

## Figures and Tables

**Table 1:** Primers used in the real-time quantitative PCR analysis.

Gene	Forword Primer (5′-3′)	Reverse Primer (5′-3′)
*β-actin*	TCTGGTGATGGTTGACCCA	GGTGAAGCTGTAGCCACGCT
*sod*	GTCCGCACTTCAACCCTCA	TCCTCATTGCCACCCTTCC
*gpx1a*	AGGCACAACAGTCAGGGATT	CAGGAACGCAAACAGAGGG
*cat*	CAAGGTCTGGTCCCATAAA	TGACTGGTAGTTGGAGGTAA
*gstr*	CGATAAGAAGGAGCACCAGA	GCCATTTCAGCAGGATTGT

## Data Availability

The data presented in this study are available in article.
